# Efficient hip joint distraction using the AO large femoral distractor in treating acetabular fractures associated with marginal impaction and intraarticular incarcerated fragments

**DOI:** 10.1186/s12891-023-07143-w

**Published:** 2024-03-27

**Authors:** Mahmoud Badran, Ahmed A. Khalifa, Ali Fergany, Bahaaeldin Ibrahim, Mohamed Moustafa, Ephrem Adem, Botond Gilyen, Osama Farouk

**Affiliations:** 1 Orthopaedic Department, Assiut University Hospital, Assiut, Egypt; 2 Orthopaedic Department, Qena faculty of medicine and University Hospital, South Valley University, Qena, Egypt; 3 Department of Orthopedics and Traumatology, Faculty of Medicine, Hawassa University, Awasa, Ethiopia; 4 Department of Orthopedics and Traumatology, Mures County Emergency Hospital, Targu Mures, Romania

**Keywords:** Acetabulum fractures, Incarcerated fragment, Marginal impaction, Large femoral distractor

## Abstract

**Purpose:**

The results after acetabular fracture are primarily related to the quality of articular reduction. Using the AO large femoral distractor, incarcerated fragments can be easily removed, and marginally impacted fragments can be elevated under direct visualization without further re-dislocating the joint. The current study aimed to evaluate our early results of using the AO large femoral distractor as an assisting tool during ORIF of acetabular fractures associated with marginal impaction or intraarticular incarcerated fragments.

**Methods:**

Eighteen patients were included in this retrospective case series study diagnosed with an acetabular fracture associated with either marginal impaction injury or an intraarticular incarcerated fragment. On a usual operative table, all patients were operated upon in a prone position through the Kocher Langenbeck approach. The AO large femoral distractor was used to facilitate hip joint distraction. Postoperative fracture reduction and joint clearance were assessed in the immediate postoperative CT scans.

**Results:**

The average age of the patients was 30 ± 8.2 years; 13 (72.2%) were males. All cases had a posterior wall fracture, and it was associated with transverse fractures, posterior column fractures, and T-type fractures in five (27.8%), two (11.1%), and one (5.6%) patients, respectively. Intraarticular incarcerated fragments were present in 13 (72.2%) cases and marginal impaction in five (27.8%). Fracture reduction measured on the postoperative CT scans showed an anatomical reduction in 14 (77.8%) patients, imperfect in four (22.2%), and complete clearance of the hip joint of any incarcerated fragments.

**Conclusion:**

The use of the AO large femoral distractor is a reliable and reproducible technique that can be applied to assist in the removal of incarcerated intraarticular fragments and to ease the reduction of marginally impacted injuries associated with acetabular fractures.

## Introduction

Acetabular fractures commonly affect young adults after high-energy trauma. An isolated posterior wall fracture is the most common type, accounting for 20–35% of cases, with a reported incidence of intraarticular incarcerated fragments occurring in up to 8% of the cases [1–4]. Obtaining anatomical acetabular fracture reduction is paramount, as imperfect reduction or leaving loose fragments intraarticularly could fasten the development of post-traumatic secondary osteoarthritis [5–9].

These fractures are usually challenging to treat owing to their complex anatomy and associated injury patterns, such as the presence of joint surface impaction injuries and intraarticular incarcerated fragments; even more, the complexity is aggravated when dealing with muscular or obese patients [6, 7], so a lot of reduction techniques and tools were suggested to facilitate the management [10].

For intraarticular incarcerated fragments and in cases associated with marginal impaction injuries, it is better to treat these injuries under direct vision, which is usually tricky due to the anatomical nature and concavity of the hip joint [4, 11, 12]. Various techniques were used to facilitate this process, including hip dislocation, which could further add to the soft tissue envelop injury and cause traction on the sciatic nerve [12, 13]; some surgeons suggested hip arthroscopy [14, 15]; however, this needs special equipment, training, and could be associated with specific complications such as neuropraxia, perineal soft tissues injuries, and ankle joint pain [16, 17].

To avoid the drawbacks and limitations of the previous techniques, joint distraction had been introduced as a practical option; this could be achieved through various techniques, either by operating on a traction table; however, if such a table was not available, surgeons either use manual traction or various tools such as the AO large femoral distractor to assist joint distraction [18, 19].

The AO large femoral distractor was used to reduce various fractures at different anatomical locations, such as femoral fractures [13, 20], tibial fractures [21], and calcaneal fractures [22]. It was reported as an assisting tool during acetabular fracture surgery [18, 23].

This study aimed to evaluate and report our experience using the AO large femoral distractor as an assisting tool to obtain anatomical reduction assessed in postoperative plain radiographs, and CT scans in acetabular fractures associated with marginal impaction or intraarticular incarcerated fragments.

## Patients and methods

This was a retrospective study based on data extracted from our pelvis trauma registry at a level one trauma center of a university tertiary referral hospital. Demographic data, fracture classification according to the Letournel and Judet system, type of intervention, operative details, and postoperative outcomes were collected for all patients diagnosed with acetabular fractures and admitted to our hospital.

During the study period (between 2018 and 2021), 420 patients were identified with an acetabular fracture, which was treated surgically (of those, 65 (15.7%) patients had concomitant osteochondral impaction injuries). The operative notes were reviewed to identify patients in which femoral distractor was used; this revealed 55 (13.1%) patients. We included patients with a preoperative diagnosis of concomitant marginal impaction or intraarticular incarcerated fragments; this revealed 25 (6%) patients. After excluding patients with incomplete radiographs or CT scans (either preoperatively or postoperatively), 18 (4.3%) Patients were included for final analysis.

### Surgical technique

Although the femoral distractor is always available in the operative theater, its uses are decided during the preoperative planning. Two senior trauma surgeons specialized in acetabular and pelvis trauma surgery operated on all cases. All patients were operated on under spinal anesthesia on a usual operative translucent table (non-traction) in a prone position. The surgeon stands on the side of the affected limb and image intensifier from the contralateral side, and after proper draping, through the standard Kocher Langenbeck (KL) approach, the posterior wall and column of the acetabulum were exposed in standard fashion. After clearing the joint of soft tissue debris and hematoma, the universal distractor was applied following the description by Calafi and Routt [18].

**Fig. 1:**
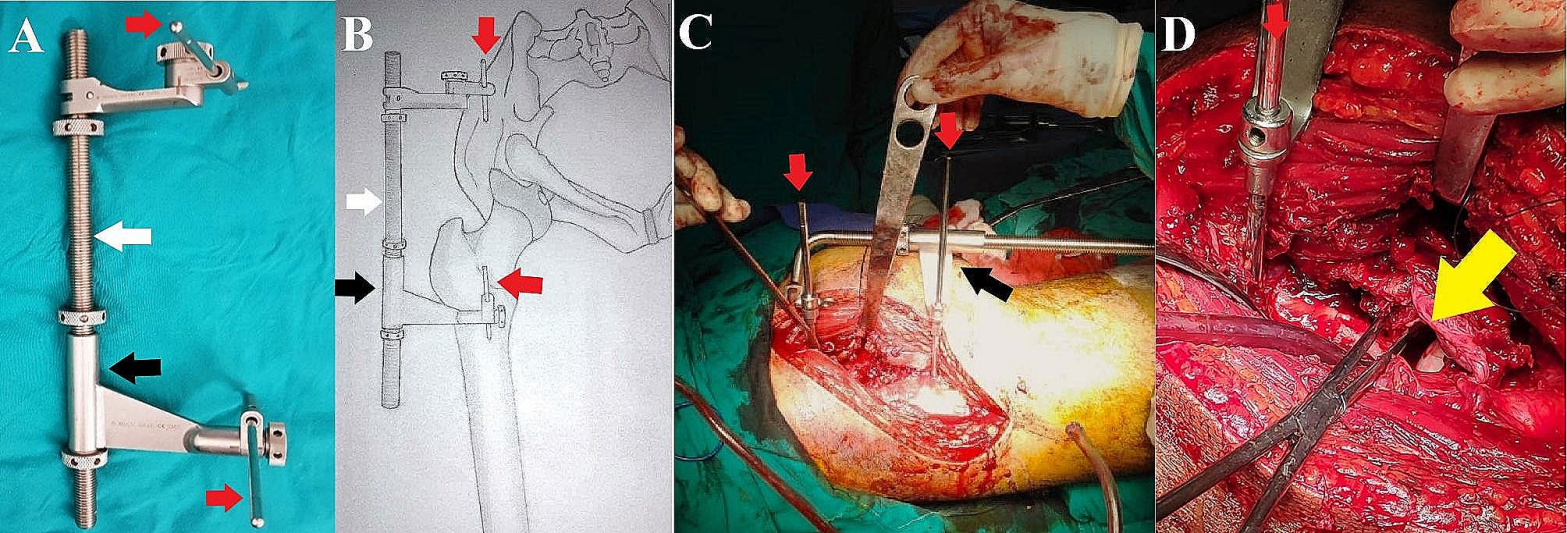
AO large femoral distractor description and clinical application. **A**, Configuration of the assembled distractor with the 330 mm threaded spindle (white arrow) and 5 mm Schanz pins in situ (red arrow). Distraction is intended caudally with the barrel of distraction placed distal (black arrow). **B**, a sketched picture of the pelvis showing the placement of the distractor across the joint in a prone position with the proximal pin on the supraacetabular dense bone and the distal pin being distal to the trochanteric area. **C**, Clinical image shows the distractor in situ assembly. **D**, The amount of hip Joint distraction (yellow arrow), which helps elevation and reduction of the impacted articular surface (held in the forceps) as well as checking the quality of reduction and achieving congruent hip joint

Two Schanz pins (5 mm) were applied, one proximally in the dense supracetabular bony area of the iliac at least two centimeters above the joint and another distally applied to the femoral shaft limited to the level of the lesser trochanter. Then, the sliding carriage was applied on the greater trochanter side for caudally directed distraction. The pins on the iliac side were tightened maximally, and the spindle nuts on both sides of the sliding carriage were turned to distract the joint. Once adequate joint distraction was achieved, ranging from 10 to 15 mm (while taking care of sciatic nerve tension first by direct palpation of the nerve and second by flexing the knee to relax the nerve), intra-articular fragments could be easily visualized and removed. In cases where a marginal impaction was present, disimpaction of the articular fragment was done under direct vision facilitated by the joint distraction, followed by anatomical reduction and bone grafting (Fig. 1).

### Postoperative quality of reduction assessment

**Fig. 2:**
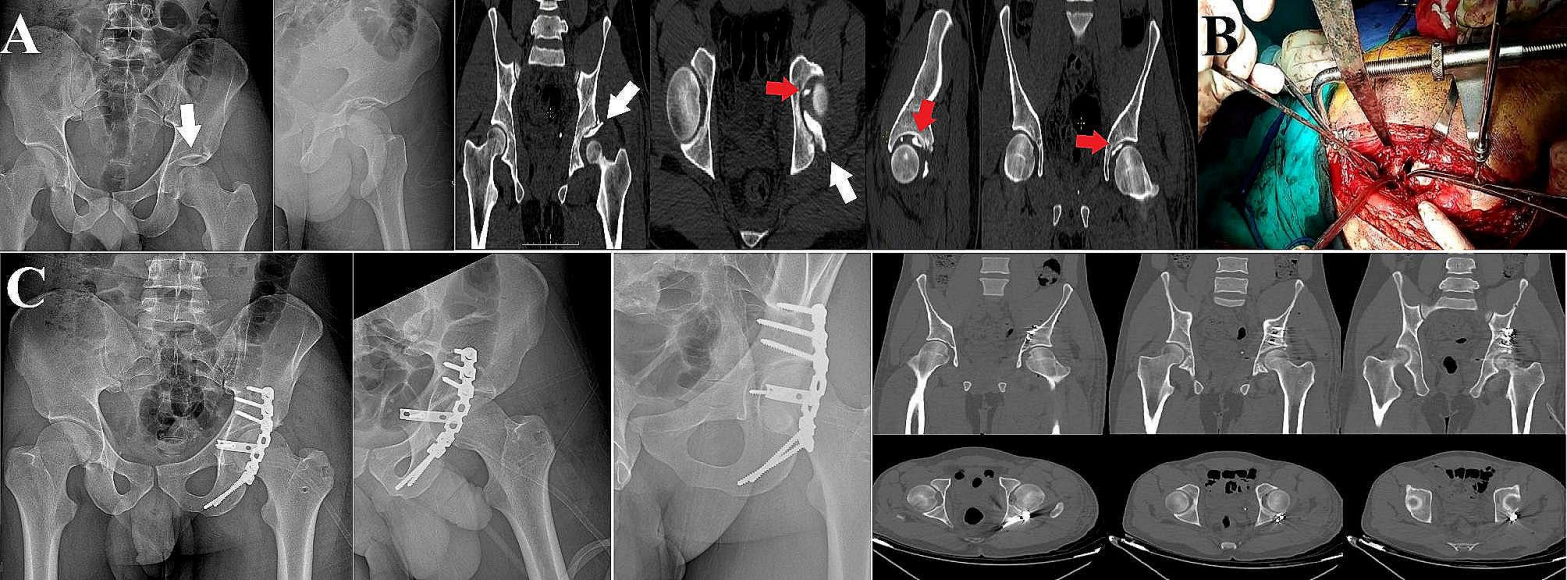
A male patient, 38 years old, presented with a posterior wall fracture of the left acetabulum. **A**, preoperative plain radiographs and CT scans showing the intraarticular incarcerated posterior wall fragment (white arrow) and a loose intraarticular fragment (red arrow). **B**, an intraoperative image showing the femoral distractor assisted hip joint distraction, which helped retrieve the posterior wall incarcerated fragment. **C**, post-operative plain radiographs and CT scans showing fracture anatomical reduction and clearance of the hip joint space

**Fig. 3:**
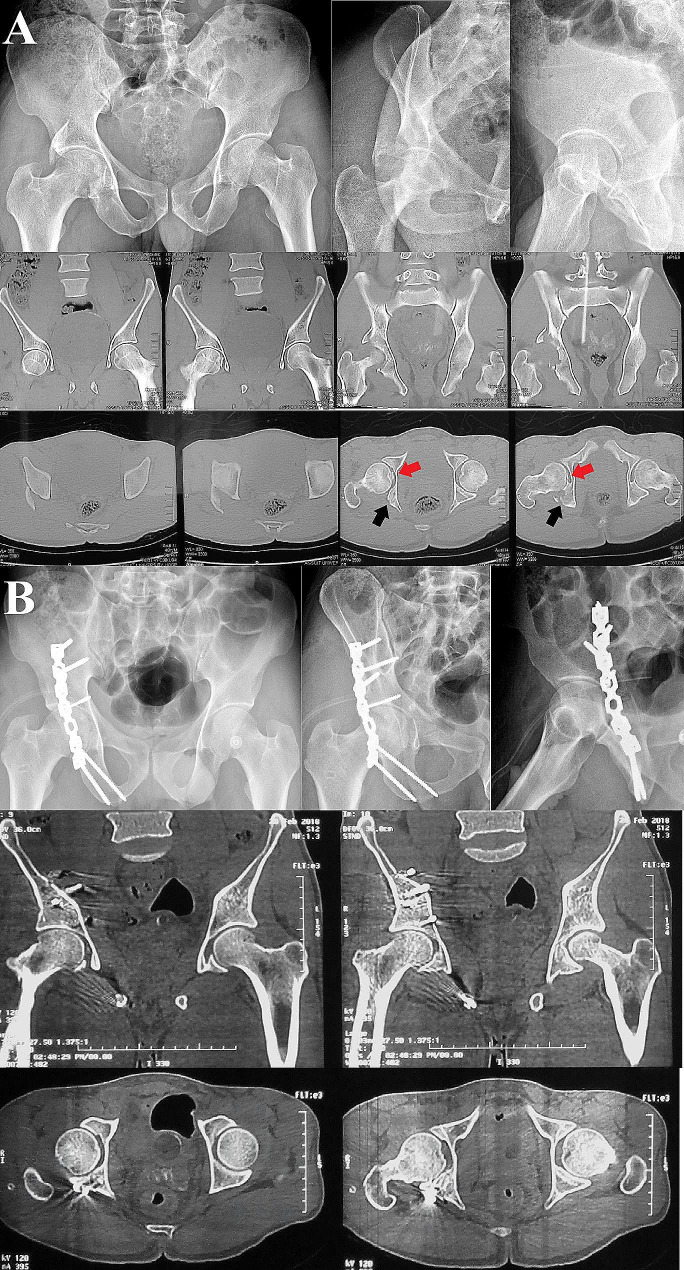
A male patient, 31 years old, presented with a posterior wall fracture of the right acetabulum associated with marginal impaction. **A**, preoperative plain radiographs and CT scans showing fracture of the posterior wall, marginal impaction injury (black arrow), and retained intraarticular loose fragment (red arrow). **B**, post-operative plain radiographs and CT scans showing fracture anatomical reduction and clearance of the hip joint space

**Fig. 4:**
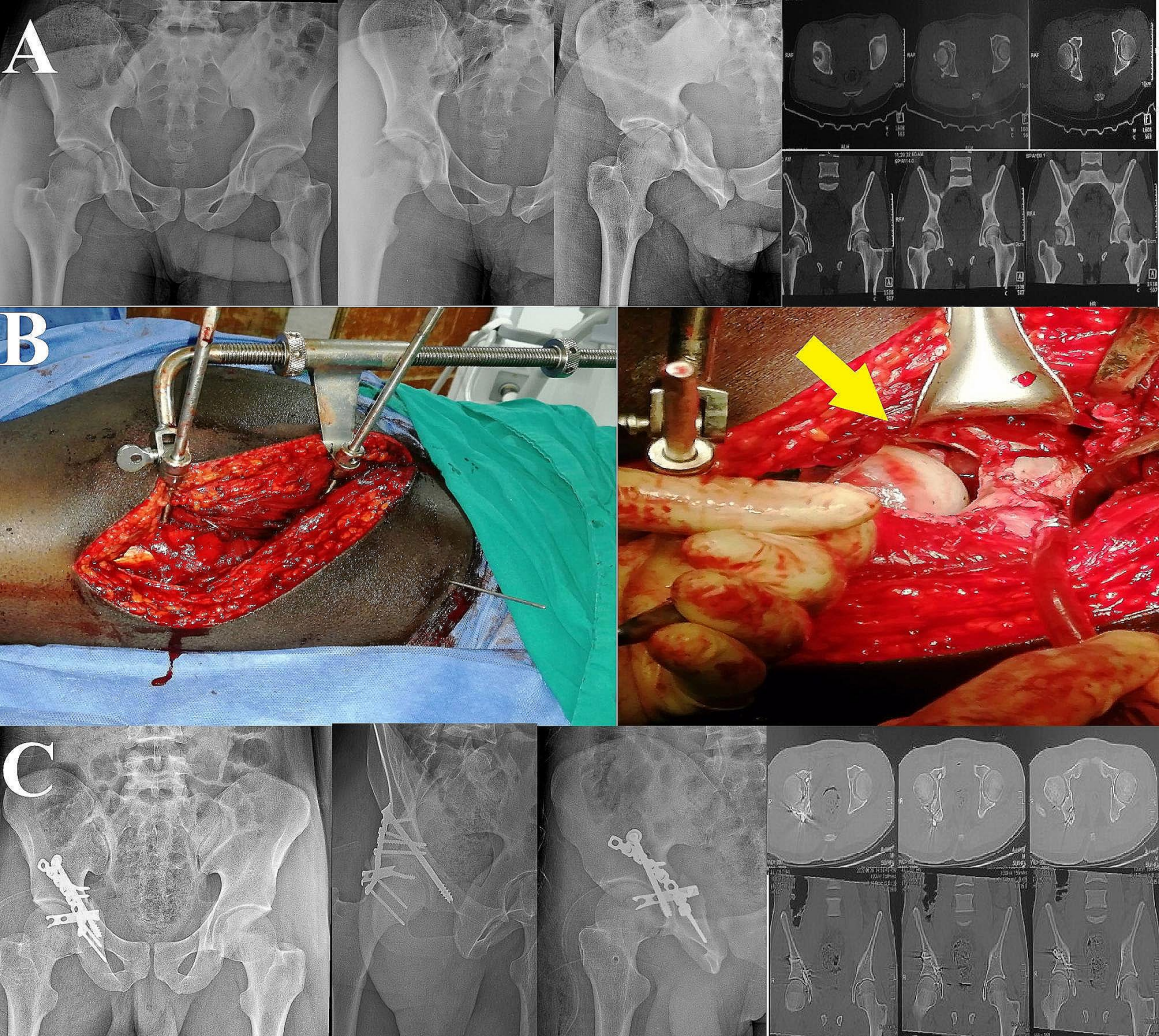
A male patient, 26 years old, presented with a Transverse fracture of the right acetabulum associated with a posterior wall fracture associated with an incarcerated intraarticular fragment. **A**, preoperative plain radiograph and CT scans. **B** shows the intraoperative application of the femoral distractor and the amount of hip joint distraction (yellow arrow). **C**, post-operative plain radiographs and CT scans showing fracture anatomical reduction and clearance of the hip joint space

In our unit, patients routinely undergo immediate postoperative CT scan assessment concomitant with plain radiographs. Three radiographic views (anteroposterior and Judet (obturator and iliac) views) were obtained, and the residual fracture displacement was measured; the reduction quality was graded according to Matta’s criteria as anatomic (0 to 1mmof displacement), imperfect (2 to 3 mm), or poor (> 3 mm) [24]. For the postoperative CT scan, the axial, sagittal, and coronal plane views were evaluated according to the method described by Verbeek et al. [25, 26] for precise assessment of the quality of fracture reduction and hip joint congruity. Furthermore, CT scans were evaluated for the presence of residual intraarticular incarcerated fragments. Radiographic and CT scan evaluations were performed by two of the authors who were not involved in the surgical procedures (Figs. 2, 3 and 4). Functional assessment was performed according to Harris Hip Score (HHS) [27] (score of 90–100 excellent, 80–89 good, 70–79 fair, and < 70 poor) and modified Merle D’Aubigné (MMD) score [28] (18 points was graded as excellent, 15–17 good, 13 or 14 fair, and < 13 as poor), we were able to collect the functional scores for 15 patients only.

#### Statistical analysis

A simple data description was provided as Means and standard deviation (range) or frequencies and percentages calculated using the Microsoft Excel program. The sample size was calculated using the statcalc program of EPI-info version 7.2 using descriptive study design calculation. Considering a population size of 65 patients, according to the previous research, the prevalence of anatomical reduction was about 80% (as reported by Giannoudis et al. [29]), with a confidence level of 90%, a degree of precision of 5%, and design effect 1, the required sample size will be 48 patients. However, the actual sample size included in the current study was lower than the required calculated sample size; this was attributed to the retrospective study nature. Furthermore, we included only patients in whom the femoral distractor was used and not all the fractures associated with an impaction injury.

## Results

**Table 1:** Patients, fractures, and management characteristics of the included group (18 (100%) patients)

*Patients basic Characteristics*		
Age	30 ± 8.2 years (range 18 to 50)	
Gender	13 (72.2%) males	
	5 (27.8%) females	
Side	11 (61.1%) right	
	7 (38.9%) left	
*Fracture classification*		
Posterior wall,	18 (100%)	
Associated transverse fracture	5 (27.8%)	
Associated posterior column fracture,	2 (11.1%)	
Associated T-type fracture	1 (5.6%).	
Associated injuries		
Intraarticular incarcerated fragments	13 (72.2%)	
Marginal impaction	5 (27.8%)	
*Management outcomes*		
Fracture reduction	anatomical	14 (77.8%)
	imperfect	4 (22.2%)
Joint clearance of incarcerated fragments	13 (100%)	

The average age of the patients was 30 ± 8.2 years (range 18 to 50); 13 (72.2%) were males, and the right side was affected in 11 (61.1%) patients. Regarding fracture classifications, all cases had a posterior wall fracture; in five (27.8%), it was associated with transverse fracture; in two (11.1%) with posterior column fracture; and one (5.6%) patient had an associated T-type fracture. Intraarticular incarcerated fragments were present in 13 (72.2%) cases, while marginal impaction was present in five (27.8%). There were no intraoperative complications related to the femoral distractor usage, and it was not aborted in any of the cases. Fracture reduction measured on the postoperative CT scans showed an anatomical reduction in 14 (77.8%) patients, imperfect in four (22.2%), and complete clearance (100%) of the hip joint of any incarcerated fragments. After an average follow up of 27.9 ± 11.1 months (range 12 to 52), the average HHS was 91 ± 8.1 (range 70 to 100), while the average MMD score was 16.4 ± 1.6 (range 13 to 18). Of the 15 patients, 14 and 13 were either excellent or good according to HHS and MMD scores, respectively.

## Discussion

In most cases, surgical treatment for acetabular fractures is needed to achieve anatomical reduction and restore the congruency and stability of the joint [2, 11]. One keystone element of the surgical technique is optimum visualization, which is usually tricky due to limited access while the hip joint is in a reduced position; this could be eased through a limited distraction of the hip joint using a large femoral distractor [18, 23].

In the current series, we obtained anatomical reduction and clearance of intraarticular incarcerated fragments (as confirmed by postoperative CT scans) while treating acetabular fractures through a Kocher Langenbeck approach with the assistance of a large AO femoral distractor without the need for a specialized traction operative table.

The direction of the initial traumatic forces and the presence of an associated hip dislocation determines the acetabular fracture complexity and further affects fractured fragments size, comminution, displacement, and the presence or absence of marginal impaction at the articular surface of the acetabulum or the femoral head [2, 10]. Furthermore, impaction injuries or incarcerated fragments could occur either during the injury incident or after the relocation of the hip joint [30, 31].

In a study by Pascarella et al. [30], the authors reported a retained intraarticular loose fragment in 45 patients out of a total of 127 patients presented with hip dislocation; the majority of the retained fragments occurred after a posterior dislocation reduction (43 out of 45 cases), the authors reported that they used two technique for removing these incarcerated fragments, either by traction through a pin inserted in the greater trochanter or dislocating the hip after manual traction applied by an assistant.

Regarding Impaction injuries associated with acetabular fractures, these could be either a dome impaction or a marginal impaction; if missed, they could lead to hip joint instability (especially if associated with posterior wall injuries) and fasten the development of post-traumatic hip osteoarthritis, so proper detection during preoperative planning and anatomical reduction of these injuries is paramount for obtaining optimum outcomes [32–34]. The incidence of marginal impaction injuries associated with posterior wall fracture could reach up to 30% [29]; furthermore, detached fragments could be incarcerated inside the hip joint and need retrieval [32]. Several management options were described to treat such injuries, including surgical hip dislocation, posterior wall osteotomy, and hip arthroscopy; however, the previously mentioned options are considered technically demanding [35–38].

In a study by Shaath et al. [23], the authors reported their results of managing 172 acetabular fractures treated through a Kocher Langenbeck approach in a prone position over five years without using a specific traction table. They reported using the universal femoral distractor among the tools used to assist fracture manipulation and reduction; they reported no malreduction of more than 2 mm in any of the cases as measured on the postoperative CT scan. The authors reported that the universal femoral distractor was used in some cases; however, they did not report precisely the indications for its use or in how many cases they used it. Furthermore, they reported that in their series, they dealt with posterior wall or posterior wall-associated patterns of fractures; however, they did not report on the presence of impaction injuries or incarcerated fragments [23]. In the current series, we decided preoperatively to use the femoral distractor after detecting either marginal impaction injuries or intraarticular incarcerated fragments as a part of preoperative planning.

We achieved hip joint clearance in all fractures associated with intraarticular incarcerated fragments, while anatomical fracture reduction was achieved in 77.8% of the cases. In a study by Giannoudis et al. [29] presented their midterm results after managing marginal impaction injuries associated with acetabular fracture. The authors reported operating while the patient was prone on a radiolucent traction table, and to obtain hip joint traction, they often used skeletal traction by a pin attached to the distal femur [29, 39]. In their series, they reported an initial anatomical reduction in 44 (73.3%) patients; however, the reduction was lost in 17 patients, leading to a final anatomical reduction of 45% [29]; indicating the challenges surgeons face when dealing with such injuries, and the need for optimum operating conditions.

Although, in the current series, we reported operating on patients in a prone position, the femoral distractor could easily be applied if the patient was in a lateral decubitus position, as reported by Calafi and Routt [18], which is attributed to the accessibility of the anatomical landmarks if the patient was in a lateral decubitus position where the two Schanz pins could be applied.

Furthermore, we reported that we used the femoral distractor in a total of 55 (13.1%) patients; in about half of those (25 patients), the indication for its use was either the presence of an intraarticular incarcerated fragment or a marginal impaction injury. However, in the remaining 30 patients, the indications were different, including assisting in the reduction of locked irreducible hip joint, in old cases where adhesions prevent appropriate fracture reduction, and in cases where the patient had a concomitant lower limb soft tissues or bony injuries where manual traction through the whole limb was not possible.

We believe that using the large femoral distractor in selected acetabular fracture cases has some advantages: First, the surgery can be performed on an ordinary fracture operative table (which is available in most institutions) without the need for a special traction table, which, if used could lead to some complications such as pudendal nerve palsy, erectile dysfunction, and perineal soft tissue injury [40, 41].

Second, some surgeons manually perform hip joint distraction or insert a lateral traction pin through the femoral neck, which is also practiced in our unit [42]. This could lead to a relatively inconsistent joint distraction and undue traction of the whole patient from the operative Table [18]. Nevertheless, constant and stable traction is preferred when dealing with complex and unstable fracture patterns, which could be applied, adjusted, and maintained using the femoral distractor.

Third, draping and preparation of the whole limb are unnecessary as the distractor applies the traction through the surgical field; therefore, this technique is helpful if the patient has a concomitant ipsilateral lower extremity soft tissue or bony injury.

Fourth, the distractor gives adequate exposure to the hip joint upon surgical manipulation and will not obstruct the view of the hip during intra-operative imaging [43]. Furthermore, as the distractor threaded spindle is attached by two arms to the Schanz screws barrels, it actually offsets the surgical field, and the surgeon could move the whole distractor away from the surgical field by applying longer Schanz pins. We did not encounter obscured visualization in the current series while the distractor was in place.

Fifth, in specific injuries such as marginal impaction, enough and maintained distraction enables the surgeon to visualize and reduce the impacted fragment and bone grafting. Furthermore, if an associated column fracture and the distractor caused displacement or obscure the column fracture reduction, the surgeon can undo the distraction until securing the column fracture and then reapply the distraction if needed.

Last, this technique does not need much of a learning curve and is practiced by most orthopedic trauma surgeons, unlike other surgical approaches used for managing impaction injuries, such as surgical hip dislocation, besides the availability of the distractor in most trauma surgery units [19, 44, 45].

This study has some limitations; first, the small sample size could be attributed to the high selectivity of the included patients and those excluded due to inadequate documents. Second, the retrospective and non-comparative nature of the study could not enable us to compare other techniques used for managing such fractures. Third, we did not report the amount of distraction performed in each case; however, this issue was extensively reported in hip arthroscopy literature. Fourth, although we did not face such issues, using a femoral distractor might be unsuitable in patients with osteoporotic bone or with metal hardware around the hip. Lastly, we should have reported on functional outcomes at each follow up visit for all patients or the long-term sequel of managing these cases.

## Conclusion

Using the AO large femoral distractor to create a controlled hip joint distraction during acetabular fracture surgery, incarcerated fragments can be easily removed under direct visualization without further re-dislocating the joint; furthermore, the elevation of marginally impacted fragments is easily facilitated with this technique.

## Data Availability

All the data related to the study are mentioned in the manuscript; however, the raw data are available with the corresponding author and will be provided upon a written request.
